# R-Wave Singularity: A New Morphological Approach to the Analysis of Cardiac Electrical Dyssynchrony

**DOI:** 10.3389/fphys.2020.599838

**Published:** 2020-12-22

**Authors:** Ping Zhan, Tao Li, Jinlong Shi, Guojing Wang, Buqing Wang, Hongyun Liu, Weidong Wang

**Affiliations:** ^1^Medical Innovation Research Division, Research Center for Biomedical Engineering, Chinese PLA General Hospital, Beijing, China; ^2^Key Laboratory of Biomedical Engineering and Translational Medicine, Ministry of Industry and Information Technology, Beijing, China; ^3^Medical Innovation Research Division, Chinese PLA General Hospital, Beijing, China; ^4^Department of Medical Engineering, Medical Support Center, Chinese PLA General Hospital, Beijing, China

**Keywords:** R-wave singularity, Lipschitz exponent, cardiac electrical dyssynchrony, ventricular dyssynchrony, autonomic nervous system, heart rate variability, acute myocardial infarction

## Abstract

R-wave singularity (RWS) measures the intermittence or discontinuousness of R waves. It has been broadly used in QRS (QRS complex of electrocardiogram) detection, electrocardiogram (ECG) beats classification, *etc*. In this article, we novelly developed RWS to the analysis of QRS morphology as the measurement of ventricular dyssynchrony and tested the hypothesis that RWS could enhance the discrimination between control and acute myocardial infarction (AMI) patients. Holter ECG recordings were obtained from the Telemetric and Holter ECG Warehouse database, among which database Normal was extracted as normal controls (*n* = 202) and database AMI (*n* = 93) as typical subjects of autonomic nervous system dysfunction and cardiac electrical dyssynchrony with high risk for cardiac arrhythmias and sudden cardiac death. Experimental results demonstrate that RWS measured by Lipschitz exponent calculated from 5-min Holter recordings was significantly less negative in early AMI and late AMI than that in Normal subjects for overall, elderly, and elderly male groups, which suggested the heterogeneous depolarization of the ventricular myocardium during AMI. Receiver operating characteristic curve analyses show that combined with heart rate variability parameters, Lipschitz exponent provides higher accuracy in distinguishing between the patients with AMI and healthy control subjects for overall, elderly, elderly male, and elderly female groups. In summary, our study demonstrates the significance of using RWS to probe the cardiac electrical dyssynchrony for AMI. Lipschitz exponent may be valuable and complementary for existing cardiac resynchronization therapy and autonomic nervous system assessment.

## Introduction

Holter electrocardiogram (ECG) is currently the only non-invasive cardiac electrophysiological monitoring tool that can provide insights into the dynamics of cardiac electrical activity during 24 h recording. Researches based on Holter ECG can be divided into two main categories: time series analysis and morphology analysis. In particular, time series such as RR intervals (RRI) and QT intervals extracted from ECG waveform are significant data sources for the analysis of autonomic nervous system (ANS) activity. However, the clinical application of Holter ECG recordings has been largely limited to detecting arrhythmic episodes and/or ectopic beats and assessing heart rate variability (HRV) (Immanuel et al., 2016). HRV is widely used to evaluate the sympathovagal balance of ANS and identify health impairment in the fact that it is cost-effective to adopt and it is easy to acquire data (Catai et al., 2019), while it provides little information about cardiomyocyte function. With the increasing popularity of Holter ECG examination, it is of great clinical significance to further improve the application value of this technology so as to expand the diagnosis scope.

Electrocardiogram morphology contains a wealth of information related to the dynamics of depolarization and repolarization that could also have significant clinical utility. QRS complex of ECG recordings reflects the electrophysiological processes of the depolarization phase. Many previous studies have consistently shown that QRS morphology is one of the most important predictors of cardiac abnormality (Zareba et al., 2011). Although changes in the repolarization phase (ST-T) are most widely used to detect acute myocardial ischemia, the detection technology of ST segment (the segment between the end of QRS complex and the onset of T wave) is far less mature than that of QRS complex. The main reason is that the shape of ST segment is diverse, which is easily affected by baseline drift and other interferences. It is difficult to identify the onset and end points of ST segment. In addition to the primary ST segment changes caused by myocardial ischemia or infarction, attention should also be paid to the differential diagnosis of other disease factors such as hypertension and autonomic dysfunction. On the other hand, it’s reported that changes also occur in the depolarization phase (i.e., the QRS complex) of the ECG during acute ischemia that could add information beyond the ST-T analysis (Romero et al., 2011). Elibet insists on separating depolarization from ventricular repolarization especially when ischemic myocardium is mentioned (Elibet, 2018).

Prior studies have reported changes in the depolarization for patients with various cardiovascular disease, including changes in QRS amplitudes (Charlap et al., 1990), QRS duration (Carballeira et al., 2014; Khurshid et al., 2016), QRS dispersion (Turrini et al., 2001; Tereshchenko et al., 2015), QRS upward slope/downward slope (Pueyo et al., 2008) and the fragmented QRS complex (fQRS) (Zhao et al., 2015; Costa et al., 2017). The increased left ventricular mass (i.e., a longer trajectory for the electrical impulse to pass) and the slowed velocity of impulse propagation are two main factors that anatomically and functionally affect QRS duration (Ljuba, 2019). During acute myocardial infarction (AMI), the glycolysis of myocardium is enhanced and a large amount of lactic acid is produced due to ischemia and hypoxia, which results in the formation of local myocardial acidosis. On the other hand, decreased adenosine-triphosphate energy supply fails to meet the needs of myocardial metabolism. In general, the dysfunctional myocardium slowed the activation of the ventricles (ventricular depolarization) and ultimately prolongs the QRS complex duration.

Cardiac electrical dyssynchrony has gained increasing attention in the past decade as an endpoint in cardiac resynchronization therapy (CRT) (Bonomini et al., 2019). However, many studies suggest that using QRS duration as the only criterion to detect dyssynchrony may have limitations. According to CRT guidelines, only a relative small proportion of patients are suitable candidates for CRT since left bundle branch block (LBBB) is a necessary criterion for CRT in patients with chronic heart failure, except for patients without LBBB but QRS duration >150 ms. Besides, studies have shown that 20 to 50% patients with heart failure and normal QRS complex have mechanical ventricular dyssynchrony (Dohi et al., 2005; Yu et al., 2006). FQRS is another marker of heterogeneous depolarization of the ventricular myocardium (i.e., ventricular dyssynchrony) that can occur due to ischemia, fibrosis, scar, or coronary microvascular dysfunction (Ljuba, 2019). It has been suggested that the mechanism of fQRS production is caused by zigzag conduction around the scarred myocardium and has been associated with aneurysms of the ischemic area (Elibet, 2018). Despite the popularity of fQRS in coronary artery disease or primary electrical abnormalities of depolarization (Brohet, 2019), it is to be emphasized that fQRS is a nonspecific finding and should only be interpreted in the presence of pertinent clinical evidence of myocardial scar as in coronary artery disease or primary electrical abnormalities of depolarization (Das and El, 2010).

A growing body of evidence supports the interventricular or intraventricular dyssynchrony, and not QRS duration, as the principal determinant of CRT outcome in nonspecific conduction diseased patients (Bonomini et al., 2019). In this study, we developed a novel approach to the analysis of QRS morphology, termed as R-wave singularity (RWS), accompanied by a new statistical metric as the measurement of cardiac electrical dyssynchrony. We applied it to well-characterized 24-h Holter monitor recordings obtained from two very distinct clinical groups: healthy subjects and those with AMI. We then test the two main hypotheses that: (1) RWS is an useful index in distinguishing AMI patients from healthy controls; (2) the addition of R-wave morphology biomarker could improve discrimination between control and AMI combined with HRV indices.

## Materials and Methods

### Data Source and ECG Processing

**FIGURE 1:**
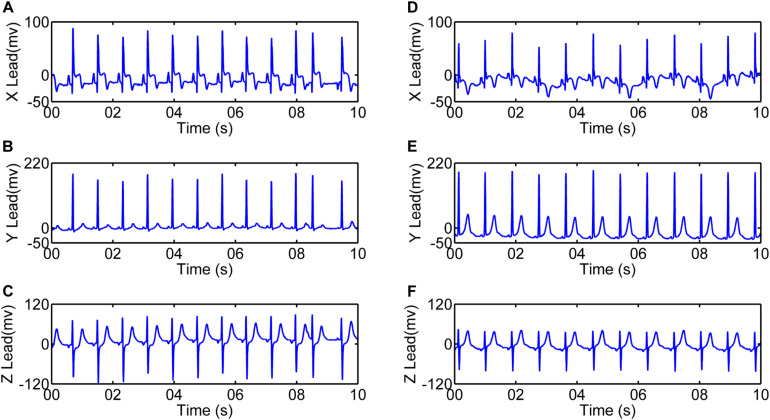
10-s electrocardiograms using three pseudo-orthogonal lead configuration (*X*, *Y*, and *Z*) for one patient with acute myocardial infarction (4041, female, 62 years old). **(A–C)** are recordings between 24 and 48 h after the event (AMI_*early*_). **(D–F)** are recordings between the 5th and 10th day after the event (AMI_*late*_).

The data for this analysis was provided by the Telemetric and Holter ECG Warehouse (THEW)^1^ at University of Rochester Medical Center, New York, United States. Database Normal (E-HOL-03-0202-003, age ranging from 9 to 82 years) from THEW contains 24-h Holter recordings of 202 healthy subjects. The other database is AMI (E-HOL-03-0160-001, age ranging from 27 to 90 years), including 24-h Holter recordings of 93 patients with acute myocardial infarction. Patients in AMI with no prespecified conditions (age, gender, treatment) were enrolled with two Holter recordings performed: one between 24 and 48 h after the event (AMI_*early*_) and the other predischarge between the 5th and 10th day after the event (AMI_*late*_). The ECG recordings were acquired using three pseudo-orthogonal lead configurations (X, Y, and Z, Figure 1). All recordings were digitized at 200 Hz. With the exclusion of those incomplete records and records with poor ECG quality (disappeared R wave or/and noise-dominant waveforms), 183 records were left in Normal, 80 records in AMI_*early*_, and 68 records in AMI_*late*_ were finally selected. Demographics of subjects included in the final analysis were summarized in Table 1.

**TABLE 1:** Demographic characteristics of overall subject groups included in final analyses.

	Normal (*n* = 183)	AMI_early_ (*n* = 80)	AMI_late_ (*n* = 68)
Age (years)	38.34 ± 15.49^a^	57.64 ± 14.64	58.87 ± 14.82
Gender	92M/91F	59M/21F	53M/15F
Body Mass Index	24.29 ± 4.56	26.86 ± 3.97	26.44 ± 3.95

*Data are expressed as mean ± SD. M for male, F for female. ^*a*^One subject -no age entry.*

**FIGURE 2:**
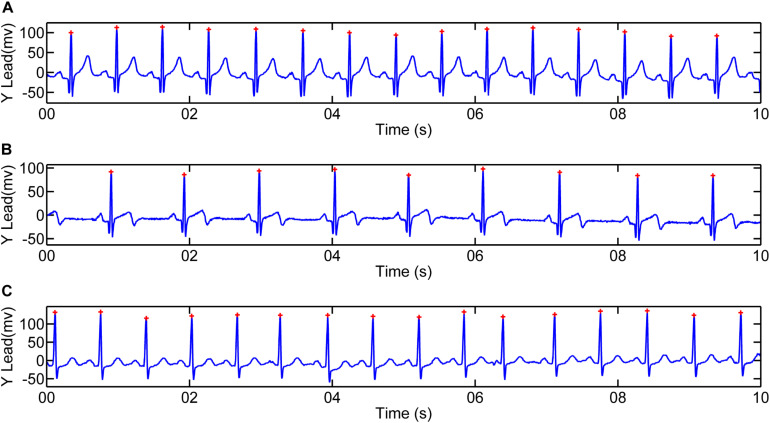
Illustration of R-wave detection for 10-s recordings. R peaks for one patient with acute myocardial infarction (4068, male, 47 years old): **(A)** Recording between 24 and 48 h after the event (AMI_*early*_); **(B)** Recording between the 5th and 10th day after the event (AMI_*late*_). **(C)** R peaks for one Normal subject (2019, male, 54 years old).

For our analysis, all 24-h Holter recordings were analyzed manually using Kubios software^2^ to extract 5-min episodes without exercise or naps within daytime (between 8 AM and 5 PM) from each ECG recording. Recordings from lead Y were then processed using the first derivative method (Friesen et al., 1990) for QRS detection in MATLAB environment (MathWorks, 2020). Figure 2 shows the detection of R waves in a patient with AMI and a normal subject. Ectopic beats were removed before further HRV analysis.

### R-Wave Singularity

**FIGURE 3:**
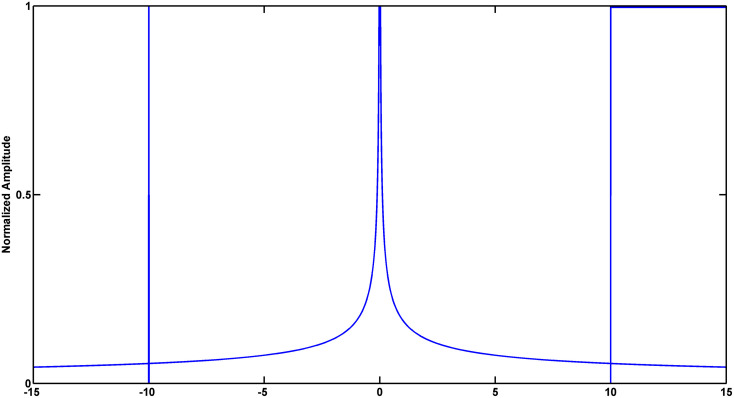
Simulated signals to illustrate the relationship between signal singularity and smoothness. From left to right: Dirac delta function, power function and step function. Exact Lipschitz exponent are −1, −0.5, and 0, respectively. Lipschitz Exponent based on wavelet transform modulus maxima (WTMM) are −1.0084, −0.4407, and 0.0100, respectively.

Signal singularity refers to the intermittent points or discontinuous derivative of the signal (Venkatakrishnan et al., 2012). The singularities contain lots of important information, which reflects the intrinsic feature or local abnormality of the signals. Lipschitz exponent (LE) is the most popular measure of the singularity characteristics of a signal. Figure 3 shows different singularities of three simulated signals (Dirac delta function, power function and step function). Noticeably, the larger LE (less negative) is, the smaller the singularity is, the smoother the signal is, and vice versa. In the case of abnormal depolarization such as ventricular desynchrony in AMI, the slowed velocity of impulse propagation makes QRS complex wider and smoother. The latter results in smaller singularity (i.e., less negative in LE).

Mallat and Hwang proved that LE can be measured by the maximum slope of straight lines that remain above the wavelet transform modulus maxima (WTMM) curve (Mallat and Hwang, 1992). Wavelet analysis deals with expansion of functions in terms of a set of basis functions. Unlike Fourier analysis, wavelet analysis expands functions not in terms of trigonometric polynomials but in terms of wavelets, which are generated in the form of translations and dilations of a fixed function called the mother wavelet (Addison, 2005). Wavelet analysis is a powerful tool for the statistical description of non-stationary signals in that the wavelet functions are localized in time and frequency and wavelet zoom is very good at localization of singularities (Venkatakrishnan and Sangeetha, 2014). This work calculated WTMM to realize the approximate measurements of LE. The validity of the algorithm was verified by simulation (Figure 3). Detailed description of this method was provided in the Supplementary Material (part 1).

Signal singularity analysis is applied in various areas such as identifying brain abnormalities (Venkatakrishnan and Sangeetha, 2014), machinery health monitoring (Kim et al., 2014), QRS detection (Tadejko and Rakowski, 2010; Wu and Bai, 2012), ECG beats classification (Daamouche et al., 2012) and so on. In this article, we calculate LE of 5-min ECG signals in R-peaks as the measurement of ventricular dyssynchrony and explore the performance of LE in distinguishing the AMI patients from the control group. Moreover, QRS durations (dQRS) were computed in overall groups to in contrast with the results of LE as the measure of dyssynchrony. Details were included in the Supplementary Material (part 2).

### Heart Rate Variability

Heart rate variability (HRV) refers to the small fluctuation of successive heartbeat intervals of sinus rhythm, affected by both sympathetic and vagal tone modulation (Riganello et al., 2012). As known to all, HRV contains lots of information about cardiovascular regulation, mainly reflecting the role of the ANS in regulating heart rate. At present, HRV analysis is mainly focused on three common approaches: standard time domain, frequency domain, and non-linear analyses. Some accompanying metrics have been used as dynamical biomarkers of cardiac vagal tone modulation (Force, 1996; Liu et al., 2018). However, HRV can be influenced by multiple factors such as respiration, blood pressure, and physical activity. Besides, there are some limits for HRV analysis such as stationary or/and linear signals. In the present study, we applied the HRV analysis based on both time domain and frequency domain analyses.

For time-domain analysis, the following parameters were taken into account: mean value of RRI (RRI, ms), the standard deviation of the mean of all sinus rhythm R-R intervals (SDNN, ms), square root of the mean of the squared differences between successive R-R intervals (root-mean-square successive difference, rMSSD) and percentage of differences between successive R-R intervals that are greater than 50 ms on overall normal beats (pNN50, %). For frequency-domain analysis, autoregression (AR) model was used for power spectrum density estimation (Zhan et al., 2016). The following parameters were calculated: low frequency (0.04–0.15 Hz) spectral power (LF); high frequency (0.15–0.40 Hz) spectral power (HF); and LF/HF ratio (LF/HF). Detailed description of this method was provided in the Supplementary Material (part 3).

Since the mean age for AMI subjects is more than 50 and aging can influence cardiac autonomic function, we selected elderly (≥40 years old) subjects for Normal and AMI groups and expected to observe statistically significant differences in LE. Furthermore, since gender can be another factor influencing the measurements of HRV, we divided each elderly group into two groups by gender. Considering that patients enrolled in group AMI_*early*_ were performed with Holter recordings between 24 and 48 h after the event and thus the drug (such as β-blockers) effect was likely to sustain by the time, the receiver operating characteristic (ROC) curve analysis in our work was limited to Normal and AMI_*late*_ subjects.

### Statistical Analysis

All statistical analyses were performed using SPSS version 20 software package (SPSS, Chicago, IL, United States). Data were presented as mean ± standard deviation (SD) for continuous variables. The differences of LE and HRV indices between AMI and Normal groups were tested using Mann–Whitney *U* test. To test the ability of LE and HRV indices to differentiate the AMI patients from the healthy control subjects and verify the hypothesis: LE could improve the discrimination accuracy of traditional HRV indices, ROC curve was constructed from the sensitivity and specificity. Binary logic regression analysis was applied to obtain the predicted probability of multivariate indices. The area under the ROC curve (AUC) gave an estimate of the overall discriminate ability. Statistical significance was accepted at the level of *p* < 0.05.

## Results

### Changes of Cardiac Dynamics in Overall Population

Traditional HRV and RWS indices of 5-min recordings for overall subjects were reported in Table 2. For AMI_*early*_ group, there was a significant increase in the mean values of RRI and LE, a significant decrease in SDNN, RMSSD, pNN50, LF, HF, and LF/HF compared with Normal group (all *p* < 0.001). For AMI_*late*_ group, there was a significant increase in the mean values of RRI and LE, a significant decrease in SDNN, RMSSD, pNN50, LF, and HF, a trend for a decrease in LF/HF compared with Normal group.

**TABLE 2:** Heart rate variability indices and Lipschitz exponent in Normal and AMI groups.

Index		Normal (*n* = 183)	AMI_early_ (*n* = 80)	AMI_late_ (*n* = 68)
Time	RRI (ms)	773.0 ± 122.6	842.4 ± 142.3**	896.9 ± 134.2**
Domain	SDNN (ms)	56.56 ± 77.93	30.63 ± 22.05**	33.12 ± 14.24**
	RMSSD (ms)	31.37 ± 32.56	18.67 ± 10.31**	20.67 ± 10.45*
	pNN50 (%)	10.23 ± 13.74	2.90 ± 4.90**	3.78 ± 6.32**
Frequency	LF (ms^2^)	362.0 ± 365.4	111.7 ± 242.9**	128.7 ± 169.1**
Domain	HF (ms^2^)	168.6 ± 288.4	47.4 ± 58.0**	54.8 ± 68.4**
	LF/HF (n.u.)	4.75 ± 4.37	3.30 ± 3.92**	4.10 ± 4.27
R-wave morphology	LE (n.u.)	−1.400 ± 0.43	−1.045 ± 0.75**	−1.102 ± 0.50**

Data are expressed as mean ± SD. *p < 0.05, **p < 0.001, AMI vs. Normal.

### Changes of Cardiac Dynamics Indices With the Subjects’ Age

The results of HRV and LE based on 5-min recordings for elderly subjects (≥40 years old) in Normal and AMI group were given in Table 3. We noticed that the significances observed in the overall study population disappeared in RMSSD for AMI_*early*_, RMSSD, and pNN50 for AMI_*late*_. However, a trend in LF/HF turned into a significance for AMI_*late*_. Nevertheless, LE with elderly subjects for both AMI groups remained significantly less negative than that in healthy group.

**TABLE 3:** Heart rate variability indices and Lipschitz exponent for elderly subjects in Normal and AMI groups.

Index		Normal (*n* = 82)	AMI_early_ (*n* = 75)	AMI_late_ (*n* = 65)
Time	RRI (ms)	769.1 ± 109.67	847.8 ± 143.59*	903.7 ± 129.90*
Domain	SDNN (ms)	42.06 ± 21.16	31.13 ± 22.53*	33.50 ± 14.32*
	RMSSD (ms)	22.81 ± 16.21	18.97 ± 10.41	20.97 ± 10.42
	pNN50 (%)	5.34 ± 9.66	3.04 ± 5.02*	3.88 ± 6.43
Frequency	LF (ms^2^)	255.40 ± 319.52	115.70 ± 250.28*	130.40 ± 171.43*
Domain	HF (ms^2^)	111.93 ± 317.45	48.48 ± 59.20*	55.78 ± 69.35*
	LF/HF (n.u.)	5.47 ± 4.97	3.29 ± 3.98*	3.88 ± 4.01*
R-wave morphology	LE (n.u.)	−1.354 ± 0.47	−1.078 ± 0.74*	−1.089 ± 0.46*

Data are expressed as mean ± SD. *p < 0.05, AMI vs. Normal.

### Changes of Cardiac Dynamics With the Subjects’ Gender

**FIGURE 4:**
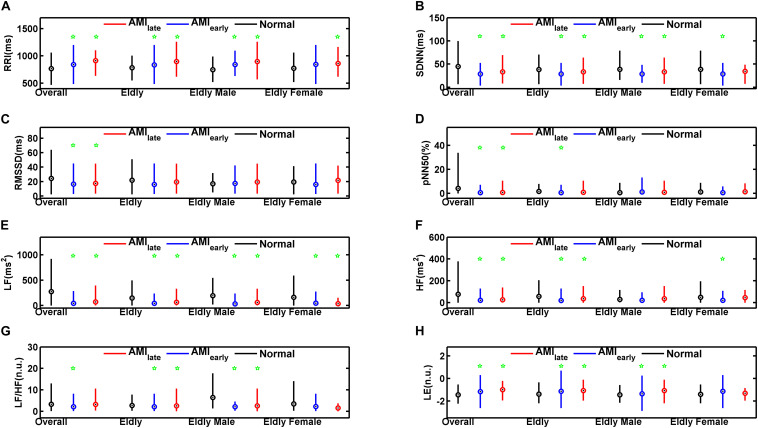
Cluster box plots of the heart rate variability and Lipschitz exponent indices for overall, elderly, elderly male, and elderly female groups of healthy subjects (Normal) and patients with acute myocardial infarction (AMI_*early*_ and AMI_*late*_), derived from the analysis of the 5-min period. The green pentagrams mark the significances (*p* < 0.05) between AMI patients and Normal in overall (Overall), elderly (Elderly), elderly male (Elderly Male), and elderly female groups (Elderly Female), respectively. RRI **(A)**: mean value of RR Intervals; SDNN **(B)**: the standard deviation of the mean of all sinus rhythm RRI; RMSSD **(C)**: square root of the mean of the squared differences between successive RRI; pNN50 **(D)**: percentage of differences between successive RRI that are greater than 50 ms on overall normal beats; LF **(E)**: low frequency (0.04–0.15 Hz) spectral power; HF **(F)**: high frequency (0.15–0.40 Hz) spectral power; LF/HF **(G)**: LF/HF ratio; LE **(H)**: Lipschitz exponent.

Tables 4, 5 show the HRV and RWS indices of 5-min recordings for elderly male group and elderly female group, respectively, of healthy and AMI subjects. The significances of pNN50 and HF further disappeared for elderly male subjects in AMI_*early*_ group, HF for elderly male subjects in AMI_*late*_ group (all *p* > 0.05, Table 4). There were few significant differences left for elderly female subjects (*p* < 0.05, Table 5) except for SDNN, LF and HF in AMI_*early*_, RRI and LF in AMI_*late*_. Figure 4 is the cluster box plots of overall summary from Tables 2–5.

**TABLE 4:** Heart rate variability indices and Lipschitz exponent for elderly male subjects in Normal and AMI groups.

Index		Normal (*n* = 36)	AMI_early_ (*n* = 54)	AMI_late_ (*n* = 50)
Time	RRI (ms)	755.8 ± 115.1	850.6 ± 150.3*	911.17 ± 122.01*
Domain	SDNN (ms)	41.08 ± 16.20	29.81 ± 19.39*	34.11 ± 15.43*
	RMSSD (ms)	19.30 ± 10.19	18.27 ± 10.06	20.44 ± 10.71
	pNN50 (%)	3.58 ± 6.19	2.73 ± 4.91	3.91 ± 6.94
Frequency	LF (ms^2^)	250.84 ± 206.77	112.80 ± 263.91*	149.71 ± 188.02*
Domain	HF (ms^2^)	48.29 ± 49.54	46.63 ± 57.59	54.84 ± 74.36
	LF/HF (n.u.)	7.45 ± 4.75	3.59 ± 4.46*	4.33 ± 4.14*
R-wave morphology	LE (n.u.)	−1.355 ± 0.52	−1.032 ± 0.74*	−1.037 ± 0.45*

Data are expressed as mean ± SD. *p < 0.05, AMI vs. Normal.

**TABLE 5:** Heart rate variability indices and Lipschitz exponent for elderly female subjects in Normal and AMI groups.

Index		Normal (*n* = 46)	AMI_early_ (*n* = 21)	AMI_late_ (*n* = 15)
Time	RRI (ms)	779.8 ± 105.33	840.6 ± 128.02	878.6 ± 155.32*
Domain	SDNN (ms)	42.84 ± 24.51	34.53 ± 29.40*	31.46 ± 9.91
	RMSSD (ms)	25.56 ± 19.36	20.77 ± 11.30	22.74 ± 9.50
	pNN50 (%)	6.71 ± 11.56	3.84 ± 5.35	3.76 ± 4.53
Frequency	LF (ms^2^)	258.96 ± 387.92	123.18 ± 217.04*	66.02 ± 69.61*
Domain	HF (ms^2^)	161.74 ± 416.79	53.24 ± 64.39*	58.92 ± 51.20
	LF/HF (n.u.)	3.92 ± 4.62	2.50 ± 2.24	2.39 ± 3.21
R-wave morphology	LE (n.u.)	−1.354 ± 0.43	−1.200 ± 0.72	−1.25 ± 0.47

Data are expressed as mean ± SD. *p < 0.05, AMI vs. Normal.

### AUCs of Cardiac Dynamics and Their Predicted Probabilities

**FIGURE 5:**
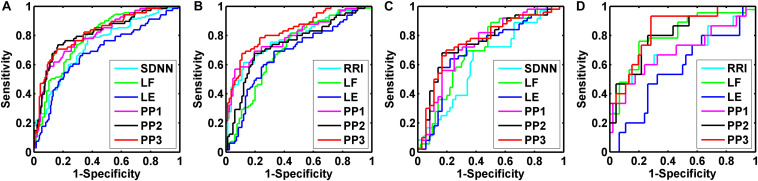
Receiver operating characteristic (ROC) curve analysis for the discrimination power of healthy subjects (Normal) and acute myocardial infarction patients (AMI_*late*_). Overall group **(A)**: the best performance of areas under the curve (AUC) was the predicted probabilities of SDNN, LF, and LE (PP3, 0.820 ± 0.031); Elderly group **(B)**: the best performance of AUC was the predicted probabilities of RRI, LF, and LE (PP3, 0.837 ± 0.033); Elderly male group **(C)**: the best performance of AUC was the predicted probabilities of SDNN, LF, and LE (PP3, 0.762 ± 0.054); Elderly female group **(D)**: the best performance of AUC was the predicted probabilities of RRI, LF, and LE (PP3, 0.838 ± 0.058).

We performed binary logic regression analysis on LE and the HRV indices which had the best AUC performance in respective domain (i.e., time domain and frequency domain) derived from the univariate index ROC curve analysis. The predicted probabilities of the time-domain index with the best AUC performance and LE (PP1), frequency-domain index with the best AUC performance and LE (PP2), and the three of them (PP3) were saved for further ROC discriminating. Figure 5 shows the results of ROC curve analysis for overall, elderly, elderly male, and elderly female subjects, respectively, in Normal and AMI_*late*_ groups.

The indices with the best AUC performance for overall subjects were SDNN for time domain and LF for frequency domain. The AUC of PP1, PP2, and PP3 were 0.804 ± 0.031, 0.816 ± 0.032, and 0.820 ± 0.031, respectively (all *p* = 0.000). For the elderly subjects, the indices with the best AUC performance were RRI and LF. The AUC of PP1, PP2 and PP3 were 0.798 ± 0.038, 0.742 ± 0.042, and 0.837 ± 0.033, respectively (all *p* = 0.000). For elderly male subjects, the indices with the best AUC performance were SDNN and LF. The AUC of PP1, PP2, and PP3 were 0.739 ± 0.056, 0.756 ± 0.054, and 0.762 ± 0.054, respectively (all *p* = 0.000). For elderly female subjects, the indices with the best AUC performance were RRI and LF. The AUC of PP1, PP2, and PP3 were 0.691 ± 0.088 (*p* = 0.027), 0.804 ± 0.065 (*p* = 0.000), and 0.838 ± 0.058 (*p* = 0.000), respectively.

## Discussion

The present study is of potential clinical application because a new method for cardiac electrical dyssynchrony quantification based on QRS morphological analysis to the short-term ECG recordings was proposed. It is the first study to show that the degree of RWS, derived from 5-min ECG recordings, grouped according to the aging process, gender classification and disease state in a cohort of healthy subjects and patients with AMI. In our study, LE proved to be a significant risk factor of AMI regardless of the onset recording time after the event. Except for the elderly female groups, LE in AMI subjects was significantly less negative than that in Normal for overall, elderly, and elderly male groups, which suggested that the singularity of R waves for patients after AMI was reduced. The reduction of RWS in AMI groups implied the existence of ventricular dyssynchrony and thus the prolonged depolarization process. Heterogeneous depolarization accounts for the abnormal behavior of ventricular muscles during the depolarization period since R waves mainly reflect the potential changes of the ventricular depolarization process. This change of QRS complex may interfere with resynchronization by causing slow, uncoordinated myocyte-to-myocyte depolarization, resulting in poor clinical outcomes in AMI patients.

R waves of ECG are the excitation process of ventricular free wall and represent ventricular depolarization in clinic. The analysis of R-wave morphology from ECG tracings recorded at different heart rates is well documented in various medical researches. For instance, poor R-wave progression (PRWP) is an important ECG finding that may be associated with many cardiac conditions, which have mortality implications for the medical director. PRWP refers to the failure to gradually increase for R wave toward V1 to V6 leads, which is commonly observed in the typical anterior myocardial infarction, LBBB, right, and left ventricular hypertrophy, Wolff-Parkinson-White syndrome and so on (Mackenzie, 2005; Kurisu et al., 2015). A prospective cohort study of 5613 healthy population (Anttila et al., 2010) suggested that PRWP was more frequent in women (7.0%) than that in men (2.7%) in three age groups 30 years or older. PRWP was an independent determinant of both all-cause (RR = 2.00, *p* = 0.002) and cardiovascular mortality (RR = 3.02, *p* = 0.001) in women, but not in men. The authors also found that the positioning of electrodes beneath rather than above the breast was not responsible for this gender-related difference. They explained that PRWP was more often associated with CHD and MI in men than that in women. Consistently, LE in women after AMI presented to be the same level as that in Normal group despite the significance in men. The decrease of RWS seems to be more relevant to male patients with AMI. Nevertheless, the small sample size of elderly female group (*n* = 21 in AMI_*early*_, *n* = 15 in AMI_*late*_) may also account for this gender-related difference.

The AUCs results showed that combined with one time-domain index and one frequency-domain index, LE evidently improved the discrimination effect of AMI in overall, elderly, elderly male, and elderly female groups. We believe that unlike HRV parameters, RWS is an effective and complementary indicator of heterogeneous depolarization, which reflects the intrinsic function of ventricular muscles and may predict the risk of myocardial necrosis, conduction block, etc., before the occurrence of myocardial infarction. Although AUCs of LE were unsatisfying and smaller than that for single HRV parameters, LE was superior in identifying pathological difference in the normalized age and gender groups (Tables 3–5).

Sympathovagal status can be influenced by various physiological and pathological factors (age, gender, health status, tobacco, medicine, circadian rhythms, noise, temperature, recording method, sampling frequency, recording period length, and removal of artifacts), of which age and gender are considered as a major determinant of HRV (Iwona and Wojciech, 2013). Therefore, the measurement of HRV should be applied under standard conditions and it is important to clinically characterize the individuals evaluated (Catai et al., 2019). In the current study, SDNN, RMSSD, pNN50, LF, HF, and LF/HF in AMI_*early*_ and AMI_*late*_ were significantly lower than those in the Normal for overall groups. A decrease in HRV has been consistently reported in patients after myocardial infarction, and contributed to both structural changes of the left ventricle and decreases in vagal activity or blunted responses of the sinus node to autonomic regulation (Casolo et al., 1992; Iwona and Wojciech, 2013). On the other hand, the increased mean RRI (i.e., decreased mean heart rate) in AMI patients revealed the severe impairment of cardiac function for such patients, which implied the myocardial damage other than ANS dysfunction. In accordance, RWS in AMI patients was significantly less negative than that in normal subjects. However, the significance of decreased HRV became vague in the case of elderly, elderly male and elderly female groups. Especially in the elderly female groups, there were no differences observed except for SDNN and LF in AMI_*early*_, RRI, and LF in AMI_*late*_.

Various studies in the cardiology area have shown the importance of HRV analysis as a tool to assess patients after myocardial infarction, patients with ventricular dysfunction, arrhythmias, to predict SCD and so on (La Rovere et al., 2003; Guzzetti et al., 2005; Neves et al., 2012). Despite the widespread application of HRV, linear approaches of HRV may be deficient and even introduce intrinsic computational errors since the regulation of the ANS on cardiac activity is considered to be nonlinear (Lombardi, 2000). We found that as the factors (age and gender) being considered, time-domain indices such as RMSSD and pNN50, failed to separate the AMI patients (Tables 3–5) from healthy subjects, while frequency-domain indices remained significant (Tables 3, 4). We also found that SDNN and LF among these HRV indices presented to be the most reliable and reproducible index for time domain and frequency domain, respectively (Tables 3–5). The reason for this finding may lie in that, SDNN reflects the overall variability of heartbeats and LF is believed to reflect both sympathetic and vagal influence correlated with baroreflex sensitivity (Anttila et al., 2010). Our study indicated the significance of the overall effect of sympathetic and vagal modulations.

The gold standard for detailed assessment of right ventricle or left ventricle dyssynchrony is still being discussed (Jurak et al., 2017). CRT guidelines emphasize that the patients undergoing CRT must have a wide QRS complex on ECGs, preferably with complete LBBB (Zhao et al., 2015). However, the identification of those patients who would respond favorably is still uncertain. Computer modeling has shown that the typical LBBB pattern can be a manifestation of slowed conduction velocity in the working myocardium, even when there is no block in the conduction system (Bonomini et al., 2019). Therefore, exploring the correlation between QRS morphological changes and ventricular dyssynchrony can help construct new, noninvasive dyssynchrony biomarkers and provide new, reliable criterion to CRT.

### Limitations

Despite the promising results in this study, there are several limitations. In our study, the results of QRS duration and RWS were not statistically compared. The significance of AUCs between two indices can be obtained by using the classical method proposed by Hanley and McNeil in 1983 (Hanley and McNeil, 1983). However, this parameter method is cumbersome to calculate the coefficient *r* between the average area and the average correlation coefficient. Anyway, the AUCs of LE and it’s optimal combination with HRV parameters (PP1, PP2, and PP3) showed greater trends compared with those of QRS duration. It can be concluded that LE is not inferior to QRS duration in terms of distinguish the two groups. In addition, we have not taken into account some confounding factors that could influence our results. For example, we have not separated groups based on the use of beta-blockers for the lack of sample size though the influences of gender and age have been taken into account in our analyses.

## Conclusion

The present findings demonstrate that the proposed new QRS morphological R-wave singularity measured by Lipschitz exponent is an effective descriptor of cardiac electrical dyssynchrony. Lipschitz exponent in AMI subjects was significantly less negative than that in Normal for overall, elderly, and elderly male groups, suggesting the existence of ventricular dyssynchrony and thus the prolonged depolarization process. Furthermore, LE is useful in improving the discrimination ability between healthy subjects and patients with AMI combined with HRV indices, which might provide valuable and complementary information for CRT, noninvasive ANS assessment, risk stratification, and efficacy prediction techniques for cardiovascular diseases. Further researches should be conducted to validate these preliminary assumptions.

## Data Availability Statement

Publicly available datasets were analyzed in this study. This data can be found here: http://thew-project.org/databases.htm.

## Author Contributions

PZ and TL conceived and designed the experiments, and analyzed the clinical data. JLS directed the statistical analysis. GJW collected the ECG data. BQW was responsible for the programming of R-wave singularity algorithm. PZ wrote the main body of the manuscript. HYL and WDW supervised the work. All authors contributed to the article and approved the submitted version.

## Conflict of Interest

The authors declare that the research was conducted in the absence of any commercial or financial relationships that could be construed as a potential conflict of interest.

## Footnotes

1 http://thew-project.org/databases.htm

2 http://kubios.uef.fi

## Abbreviations

ECG: electrocardiogram
RRI: RR intervals
LE: Lipschitz exponent
RWS: R-wave singularity
HRV: heart rate variability
ANS: autonomic nervous system
fQRS: fragmented QRS complex
AMI: acute myocardial infarction
ROC: receiver operating characteristic
AUC: area under the ROC curve
CRT: cardiac resynchronization therapy
LBBB: left bundle branch block.
