# Supplementary material for: R-Wave Singularity: A New Morphological Approach to the Analysis of Cardiac Electrical Dyssynchrony
Source: Front Physiol. 2020 Dec 22;11:599838. doi: 10.3389/fphys.2020.599838 (PMC7783454; doi:10.3389/fphys.2020.599838)
Supplement: Supplementary file 1 [file Presentation_1.pdf]

Supplementary Material

1. R-wave Singularity

A function $f(t)$ is said to be Lipschitz α, for at a point $t = t0$, if and only if there exists a constant $t0$ such that for all points t in a neighborhood of $t0$

$$|f(t+h) - f(t)| \leq C|h|^{\alpha} \quad (1)$$

Written as $f(t) \in C_{t0}^{\alpha}$, where C is a constant and α is the LE of $f(t)$ in $t0$. Noticeably, the larger α is, the smaller the singularity is, the smoother the signal is, and vice versa.

The *n*th derivation of the Gaussian function is usually a popular wavelet in practice. Hence, we chose the second derivative of Gaussian function Mexican hat function (MHF), as the wavelet (Supplementary Figure 1.). MHF has good localization in both the time domain and frequency domain. Moreover, MHF prevents the position of the singularity from shifting for its symmetric property. MHF expressions are shown as follows

$$\Psi(t) = (1 - t^2)e^{-\frac{t^2}{2}} \quad (2)$$

$$\Psi(w) = \sqrt{2\pi}w^2 e^{-\frac{w^2}{2}} \quad (3)$$

After the wavelet was selected, the local maximum point was sought near the feature point $t0$ (i.e. R-peaks in this paper) in the range which remained constant as the $\alpha \to 0+$. Finally, the module value of the local maximum point (i.e. WTMM) was computed to estimate the $\alpha(t0)$[1].

2. QRS duration

We also computed QRS durations (dQRS) in overall groups to verify the advantages of Lipschitz exponent (LE) as the measure of dyssynchrony. Supplementary Table 1 shows dQRS of 5-min recordings for overall subjects. There was significant increase in dQRS for both AMI_{early} and AMI_{late} groups, sugggesting the existence of dyssynchrony in the AMI patients. Furthermore, receiver operating characteristic curve (ROC) analysis (Supplementary Table 2) shows the areas under the curve (AUC) of dQRS was 0.633±0.048 (smaller than that of LE, 0.686±0.039) and the best performance was the predicted probabilities of LF and dQRS (PP2, 0.790±0.034), which is also smaller than that of LE (PP3, 0.820±0.031). Hence, LE is an effective index of ventricular dyssynchrony and has a better performance in distinguishing between the AMI patients and healthy subjects.

3. Heart Rate Variability

AR model equation can be expressed by

$$x(n) = -\sum_{i=1}^{p} a_p(i)x(n-i) + w(n) \quad (4)$$

where， $w(n)$ is a white noise with a mean of zero and a variance of σ^2. p is the order of AR model. Hence, the power spectrum of AR model is estimated by

$$\boldsymbol{P_x(k)} = \frac{\boldsymbol{\sigma^2}}{|\boldsymbol{1}+\sum_{i=i}^{p} \boldsymbol{a_i W_N^{-ki}}|^2} \quad (5)$$

The model-based parameter method can effectively avoid spectrum leakage caused by a window function, thus providing better resolution, and can be more sensitive to the fast change of HRV. At the same time, it can obtain a smoother spectrum compared to fast Fourier transform (FFT), and is easy to calculate the center frequency of each frequency band, achieving accurate PSD estimation even when there is little data. Burg algorithm was used to estimate the coefficient of AR model in this study for its high-frequency resolution and improvements in the frequency bias as well as spectral line splitting[2]. The order p of the AR model is set to 16[3].

Supplementary Table 1. QRS durations (dQRS) in Normal and AMI groups.

Index	Normal (*n*=162)	AMI_{early} (*n*=65)	AMI_{late} (*n*=53)
dQRS	81.715±15.648	94.570±18.883**	90.945±20.946**
P value		0.000	0.004

Note: Data are expressed as mean ±SD. ** *p* : *p*<0.001, AMI *vs.* Normal.

Supplementary Table 2. Receiver operating characteristic curve (ROC) analysis of QRS durations (dQRS) and predicted probabilities for Normal and AMI_2 groups.

Index	AUC	P value
dQRS	0.633±0.048	0.004
PP1	0.758±0.035	0.000
PP2	0.791±0.035	0.000
PP3	0.790±0.034	0.000

Note: Data are expressed as mean ±SD. PP1 for the predicted probabilitie of SDNN and dQRS, PP2 for the predicted probabilitie of LF and dQRS, PP3 for the predicted probabilitie of SDNN, LF and dQRS.

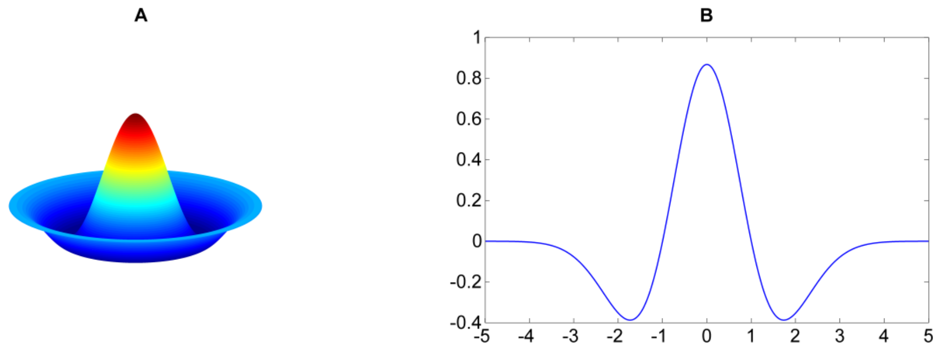

Supplementary Figure 1. Mexican hat wavelet for the three-dimensional plot (A) and two-dimensional plot (B), respectively.

References

[1] Venkatakrishnan, P., Sangeetha, S., and Sundar, M. (2012). Measurement of Lipschitz exponent (LE) using wavelet transform modulus maxima (WTMM). *International Journal of Scientific & Engineering Research* 3:6.

[2] Hu, G. S. (2003). Digital Signal Processing. Beijing: Tsinghua University Press.

[3] Boardman, A., Schlindwein, F. S., Rocha, A. P., and Leite, A. (2002). A study on the optimum order of autoregressive models for heart rate variability. *Physiological Measurement* 23, 325-336. doi:10.1088/0967-3334/23/2/308
